# Bioengineered skin organoids: from development to applications

**DOI:** 10.1186/s40779-023-00475-7

**Published:** 2023-08-22

**Authors:** Zi-Xuan Hong, Shun-Tian Zhu, Hao Li, Jing-Zhi Luo, Yu Yang, Yang An, Xi Wang, Kai Wang

**Affiliations:** 1 Department of Physiology and Pathophysiology, School of Basic Medical Sciences, State Key Laboratory of Vascular Homeostasis and Remodeling, Peking University, Beijing, 100191 China; 2 Department of Hepatopancreatobiliary Surgery, the Third Affiliated Hospital of Soochow University, Changzhou, 213000 Jiangsu China; 3 Department of Plastic Surgery, Peking University Third Hospital, Beijing, 100191 China; 4 Clinical Stem Cell Research Center, Peking University Third Hospital, Beijing, 100191 China; 5 State Key Laboratory of Cardiovascular Disease, Fuwai Hospital, Beijing, 100037 China; 6 Beijing Key Laboratory of Metabolic Disorder Related Cardiovascular Disease, Capital Medical University, Beijing, 100050 China

**Keywords:** Skin organoid, Organoid generation, Skin appendage, Tissue engineering, Disease modelling, Regenerative medicine

## Abstract

Significant advancements have been made in recent years in the development of highly sophisticated skin organoids. Serving as three-dimensional models that mimic human skin, these organoids have evolved into complex structures and are increasingly recognized as effective alternatives to traditional culture models and human skin due to their ability to overcome the limitations of two-dimensional systems and ethical concerns. The inherent plasticity of skin organoids allows for their construction into physiological and pathological models, enabling the study of skin development and dynamic changes. This review provides an overview of the pivotal work in the progression from 3D layered epidermis to cyst-like skin organoids with appendages. Furthermore, it highlights the latest advancements in organoid construction facilitated by state-of-the-art engineering techniques, such as 3D printing and microfluidic devices. The review also summarizes and discusses the diverse applications of skin organoids in developmental biology, disease modelling, regenerative medicine, and personalized medicine, while considering their prospects and limitations.

## Background

The skin, being the body’s largest organ, performs a range of functions, including protection, sensation, and thermoregulation. It comprises three layers enclosed by a membrane: the epidermis, dermis, and hypodermis. The epidermis consists of closely interconnected keratinocytes that produce a stratum corneum to withstand environmental factors. The dermis is a complex structure, housing mechanoreceptors, sensory nerves, blood vessels, sweat glands, hair follicles, as well as an abundant extracellular matrix and fibroblasts. The hypodermis contains subcutaneous adipose tissue, which stores energy and growth factors [1, 2]. The skin also houses a robust immune system, including Langerhans cells in the epidermis, dendritic cells in the dermis as part of the innate immune system, and peripheral leukocytes recruited during infection resistance [3].

The concept of organoids has evolved alongside advancements in related fields. Broadly speaking, organoids are three-dimensional (3D) cultures derived from pluripotent stem cells, fetal stem cells, or adult stem cells. In a broader sense, organoids refer to 3D cell cultures that can mimic specific features of organs or tissues in the human body. In our review, this broader definition encompasses the concepts of “cellular spheroid or aggregate”, “reconstructed 3D skin” and “bioengineered skin structure”. The skin organoids discussed in this review are in vitro 3D tissue constructs comprising various cell types and exhibiting morphological and functional competence as skin surrogates.

**Fig. 1:**
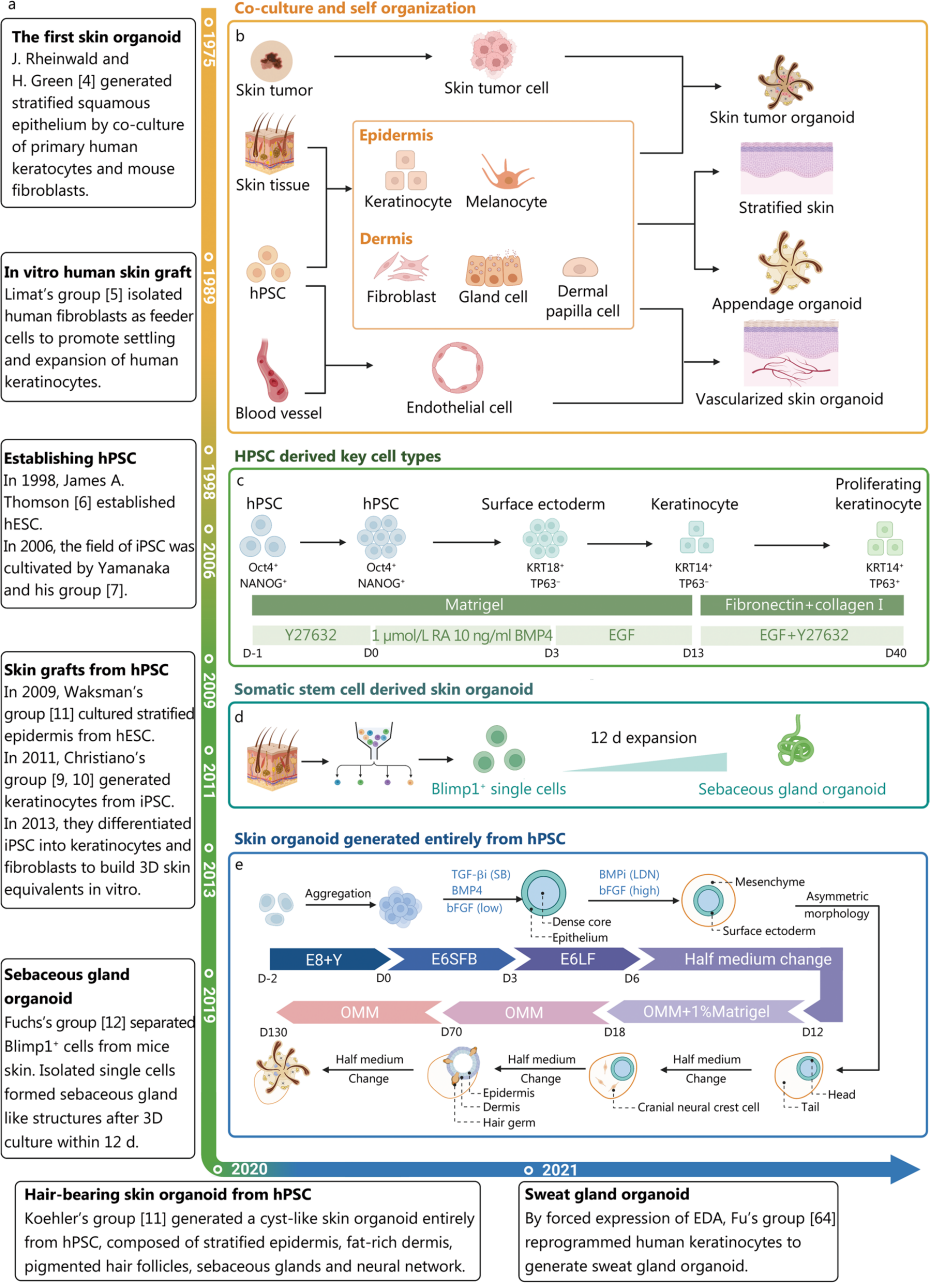
Milestones and technical roadmap of skin organoid generation. **a** Since the establishment of the first skin organoid by Rheinwald and Green in 1975, significant progress has been made in generating skin organoids, marking various milestones in this field. **b** The conventional protocol for generating skin organoids involves utilizing the self-organization ability of different cell populations. These cells can be sourced from healthy skin tissue, tissues with inherited diseases, or tumors. Additionally, human pluripotent stem cells (hPSCs) have emerged as another cell source following the development of differentiation protocols. Vascularization is also considered by incorporating human umbilical vein endothelial cells (HUVECs). **c** However, the generation of skin-specific cells from hPSCs remains a challenge. In 2011, Christiano's group successfully addressed the issue of deriving keratinocytes from hPSCs. **d** Culturing somatic stem cells is another promising approach. Fuchs et al. separated Blimp1^+^ cells from skin tissue and successfully constructed sebaceous gland organoids through a 12 d 3D culture in vitro. **e** In 2020, Lee et al. published their work on generating skin organoids entirely from induced pluripotent stem cells (iPSCs). These cyst-like structures are well-stratified and contain rich appendages. iPSC induced pluripotent stem cell, hESC human embryonic stem cell, hPSC human pluripotent stem cell, EDA ectodysplasin A, RA retinoic acid, BMP4 bone morphogenic protein 4, KRT keratin, TP63 tumor protein p63, E8 essential 8 medium, Blimp1 B lymphocyte induced maturation protein 1, bFGF basic fibroblast growth factor, E6SFB E6 medium + SB431542 + bFGF + BMP4, E6LF E6 medium + LDN + bFGF, OMM organoid mature medium

The idea of a skin culture system as an in vitro substitute was first proposed in 1975. Rheinwatd et al. [4] were pioneers in developing a self-organizing strategy for generating squamous epithelium, which involved serial co-cultivation of primary human keratocytes and irradiated mouse fibroblasts. This breakthrough paved the way for in vitro culture of self-organized skin tissue. In 1989, a fibroblast feeding strategy was introduced to ensure stable settlement and expansion of keratinocytes [5]. Subsequently, embryonic stem cells (ESCs) and induced pluripotent stem cells (iPSCs) were successively utilized as powerful and efficient tools for studying in vitro skin organogenesis. In the late 2000s and early 2010s, 3D self-organized stratified epidermal equivalents derived from ESCs and iPSCs were developed, representing a significant milestone in the field of skin organoids [6–10]. This marked a major breakthrough, establishing skin organoids as potent tools for in vitro skin culture. In 2020, Lee et al. [11] reported the construction of an almost complete in vitro self-organized skin system differentiated from iPSCs, forming a hierarchical skin organoid that recapitulated many appendage structures, including hair follicles. Almost simultaneously, organoids containing sebaceous or sweat glands derived from reprogrammed epithelial tissue cells were developed, demonstrating the integration of appendages into a mature skin generation system [12, 13] (Fig. 1a).

Skin organoids hold great promise for studying organogenesis, drug testing, and regenerative medicine. However, there are challenges related to scientific and ethical aspects that need to be addressed. These include the lack of key cell types and the inability to fully replicate the complex structure of native skin. Additionally, the long duration required for differentiation and maturation hinders their application in urgent patient needs. Furthermore, the limited size and lifespan of organoids restrict their broader application and accessibility. In this review, we aim to provide a comprehensive summary of existing studies on skin organoids, covering topics such as culture methods, maturation techniques, applications, and limitations. We believe that advancements in engineering methods and a deeper understanding of skin differentiation processes will accelerate the development of more robust and functional skin organoids [14].

## Skin structure and organogenesis

### Epidermis

The epidermis, a stratified epithelium, consists of two main cell types: the basal layer containing epidermal stem cells and the superficial layer composed of specialized keratinocytes. The epidermal stem cells are responsible for the continuous regeneration of the epidermis, which occurs every 40–56 d, as they undergo proliferation [15]. As these proliferative cells move outward, they undergo differentiation, forming distinct layers in the epidermis, including the spinous, granular, and cornified layers [16]. Recent studies have revealed the presence of heterogeneous populations of epidermal stem cells, challenging the notion that a single population is solely responsible for maintaining the skin's plasticity and homeostasis [17].

During embryonic development, the epidermis originates from the surface ectoderm, which is characterized by the expression of Keratin 8 (KRT8) and KRT18 after neurulation. The Wnt signaling pathway plays a dominant role in directing the ectodermal cells towards an epidermal fate by inhibiting the response to fibroblast growth factors (FGFs). This inhibition promotes the expression of bone morphogenetic proteins (BMPs), which in turn initiate the differentiation process leading to epidermal formation [18]. As the cells in the basal layer of the epidermis, characteristic markers such as KRT5 and KRT14 are upregulated, while KRT8 and KRT18 are downregulated [19]. As keratinocyte progenitor cells commit to terminal differentiation, they migrate to the upper basal layer and express KRT10. Above this layer, the granular layer and cornified layer are formed and marked by the presence of filaggrin and loricrin [20]. The basement membrane provides structural support to the basal epidermal cells and also plays a crucial role in signaling pathways that determine cell fate and function [21]. Melanocytes, responsible for skin pigmentation, are located in the basal layer and originate from neural crest cells [22].

### Dermis and hypodermis

The dermis, positioned beneath the epidermis's basement membrane, is a connective tissue layer that provides support to the epidermis and skin appendages. It is characterized by a rich extracellular matrix and contains blood vessels, lymphatics, nerves, adipocytes, and immune cells [23]. The dermis is divided into two distinct layers: the reticular dermis and the papillary dermis. The papillary dermis, with higher enzymatic activity and fibroblast density, plays a crucial role in hair follicle formation. On the other hand, the reticular dermis, located in the lower region, facilitates initial dermal repair and recruits cells from the former layer [24].

The dermis originates from three distinct mesenchymal sources. The neural crest contributes to the development of the dermis in the face and neck, while the lateral plate mesoderm is responsible for the dermis in the limbs and body wall. The paraxial mesoderm gives rise to the dermis in the back [2]. Fibroblasts throughout the dermis express high levels of platelet derived growth factor receptor α (PDGFRα) and PDGFRβ, which are involved in the regulation of collagen fibril assembly [25]. Interestingly, specific fibroblast markers can vary across species. In human skin, SFRP2/DPP4 and FMO1/LSP1 are used to define major fibroblast populations, while Watt’s group has identified markers for murine dermal papilla (CRABP1), papillary (DPP4/CD26), and reticular (PDPN, SCA1/ATXN1) fibroblasts [24, 26, 27].

The hypodermis, situated beneath the reticular dermis, consists of loosely arranged connective tissue. The thickness of subcutaneous adipose tissue varies depending on body location, sex, and nutritional status. In addition to its role in thermal insulation and energy storage, adipocytes contribute to the regenerative cycle of hair follicles by secreting various factors, including platelet-derived growth factor (PDGF) [28, 29].

### Skin appendages

The skin appendages encompass the pilosebaceous unit, sweat glands, and nails. The pilosebaceous unit consists of the hair shaft, hair follicle, sebaceous gland, and arrector pili muscle. The hair shaft exhibits intricate structure and diverse patterns, distinguishing beard, hair, and lanugo from one another [30–32]. The development of the hair shaft involves multiple stem cell populations and signaling pathways. Ectodermal hair follicle stem cells (HFSCs) give rise to the sebaceous gland and apocrine gland, while mesoderm-derived cells develop into the follicular dermal papilla and the connective tissue sheath. Neural crest-derived melanocyte progenitors generate the pigmentary unit located near the dermal papilla [33]. The Wnt/β-catenin pathway plays a role in the activity and cell fate decisions of skin stem cells. Asymmetrical Wnt signaling leads to the formation of the hair medulla (marked by KRT75), inner root sheath (marked by KRT71), outer root sheath (marked by KRT5), and the bulge [34]. In the absence of β-catenin, the differentiation ends with the epidermis instead of follicular keratinocytes [35]. Paracrine transforming growth factor β (TGF-β) activates SMAD2/3 to counterbalance the inhibitory signal of BMP. HFSCs express the target gene Tmeff1 to lower the BMP thresholds [36].

Mesenchymal-epithelial signaling regulates epidermal patterning and the morphogenesis of skin appendages [37]. Eccrine sweat glands express K7, K9, and carcinoembryonic antigen (CEA), with CEA being exclusively expressed in sweat glands found in normal skin [38]. Blimp1 plays a crucial role in the progenitor cells of sweat glands, and the loss of Blimp1 stimulates c-myc, which enhances bulge stem cell activity [39]. The specification of sweat glands occurs through mesenchymal-derived BMPs and fibroblast growth factors, which signal to epithelial buds and suppress epithelial-derived sonic hedgehog (SHH) production. Conversely, mesenchymal SHH outweighs BMP production in hair follicles, leading to their specification [40].

The specific identity of cells constituting the cutaneous niche responsible for skin homeostasis and regeneration remains poorly understood. Therefore, further research on the interaction and communication between different cell lineages is crucial for advancing the development of skin organoid models. Moreover, the skin exhibits variations throughout the body in terms of thickness, texture, and the presence of different structures. Certain areas of the skin, such as the palms and soles, have a thicker epidermis and lack the pilosebaceous unit, but host numerous sweat glands [2]. Therefore, the ability of skin organoids to regenerate multiple skin types in vitro is essential to accommodate the diverse characteristics of skin in distinct regions.

## Generation of skin organoid

### 3D layered skin substitute

3D in vitro skin analogues have the capability to faithfully replicate the complete structure and cellular composition of native skin, including the epidermis, dermis, and skin appendages, surpassing the accuracy and fidelity of conventional 2D models [41, 42] (Fig. 1b). In 2011, Itoh et al. [9] successfully generated 3D skin equivalents by seeding iPSC-derived keratinocytes onto a matrix scaffold containing normal human fibroblasts (Fig. 1c). The differentiation of iPSCs into keratinocytes was achieved using RA and BMP4. RA promoted epithelial differentiation, while BMP4 prevented neural differentiation [43]. The resulting organoids exhibited stratification of iPSC-keratinocytes and closely resembled the complex layering of the native epidermis [44]. Subsequently, fibroblasts isolated from normal human tissue were replaced with iPSC-derived fibroblasts, leading to the generation of purely iPSC-derived organoids [10]. Kim et al. [20, 45, 46] followed a similar approach in constructing skin organoids. They generated iPSC lines from HLA-homozygous cord blood mononuclear cells and peripheral blood mononuclear cells. Their method offered advantages in overcoming immune rejection and showed great potential for clinical use. Additionally, they introduced EGF during keratinocyte differentiation to stimulate proliferation and differentiation [47]. The team successfully transplanted their organoids into immunodeficient mice, effectively healing skin lesions.

Engineering techniques have proven effective in restoring the complex structure of human skin. Blackstone et al. [48] utilized 3D printing to recreate rete ridge-like structures, resulting in a significant improvement in basement membrane formation and promoting epidermal proliferation and differentiation. Melanocytes, responsible for skin photoprotection and thermoregulation, are typically located in the basal layer of the epidermis at the junction with the dermis [49]. The addition of iPSC-derived melanocytes to skin equivalents has increased the complexity of in vitro skin models [50]. Supp et al. [51] demonstrated that such skin models possess photoprotective abilities, reducing the damage caused by UV-induced DNA damage.

### Hair follicle organoid

The functionality and clinical applications of skin organoids have been significantly limited by the absence of skin appendages. Therefore, the regeneration of engineered skin tissue containing appendages is crucial for the development of human skin substitutes and advancements in regenerative medicine. One common approach to achieving in vitro human hair follicle formation involves combining dermal papilla cells with epithelial components. Isolated human dermal papilla cells can partially regain their inductive capability when grown as spheroids, leading to the induction of human hair follicle neogenesis [52]. Kalabusheva et al. [53] established two hair follicle germ models using human dermal papilla cells and keratinocytes. They found that the mixing of these two cell types as aggregates better represents the interactions between cells and their surrounding niche during hair follicle reconstruction compared to simply coating dermal papilla cells with keratinocytes. The researchers investigated the effects of various soluble factors and extracellular matrix components on the organoids and identified hyaluronic acid (HA) as a stimulating factor that significantly increased proliferation and aggregate size. In a similar study, the addition of hair follicle stem cells and silk fibroin to the culture system created a more complex microenvironment [54]. The organoids exhibited reduced levels of BMP signals and upregulated expression of the β-catenin gene, which are crucial for dermal papilla cell function, hair follicle differentiation, and hair cycle maintenance [35, 55]. The model also demonstrated gene expression patterns similar to those observed in vivo, indicating its resemblance to the early-stage anagen phase of hair follicle development. Both of these studies constructed cell aggregates in vitro without transplanting them into animal models to validate their functionality. Su et al. [56] created a hair follicle organoid by mixing human dermal progenitor cells with epidermal stem cells and transplanted the aggregate onto the dorsal skin of nude mice, resulting in hair formation. Their research demonstrated the potential of hair follicle organoids to generate hair in vivo as grafts. They also identified Wnt pathway activation as indispensable for hair regeneration, with LEF1 serving as a biomarker for hair regeneration due to its significant expression during the formation of hair follicle organoids. However, the use of cells derived from fetal tissues may raise ethical concerns, necessitating the substitution of non-fetal cells such as human induced pluripotent stem cells (hiPSCs).

In 2020, Lee et al. [11] achieved a significant breakthrough in hair regrowth by creating a sophisticated human skin organoid from human pluripotent stem cells (Fig. 1e). They manipulated the TGF-β, FGF, and BMP signaling pathways and co-induced surface ectoderm cells and cranial neural crest cells within PSC aggregates. After approximately 140 d of incubation under floating rotation cultural conditions, the skin organoid developed into a complex tissue consisting of stratified epidermis, pigmented hair follicles, sebaceous glands, adipocytes, Merkel cells, and sensory neurons, closely resembling the natural process of skin development in vivo [57]. The hair follicles grew radially inward in vitro and assumed their normal morphology when the cell cysts unfolded into a planar skin structure after transplantation onto nude mice. The establishment of hair-bearing skin organoids with innervation provides an ideal model for studying skin neogenesis, structure, and diseases. Several studies have validated the feasibility of using and optimizing Lee's protocol to generate skin organoids from different hiPSC lines [58, 59]. However, the long incubation period of over 140 d for in vitro skin organoids is labor-intensive and time-consuming, limiting its practical applications.

In contrast, biofabrication methods offer an attractive approach to producing large quantities of skin appendage organoids. A recent study presented a culture system for generating human dermal papilla spheroids [60]. The researchers mixed human dermal papilla cell aggregates with Matrigel and co-cultured them with hair matrix cells and dermal sheath cup cells. However, a significant limitation was the use of cell lineages isolated from human tissues, making it challenging to generate ample amounts of skin substitutes. Therefore, future investigations are expected to focus on utilizing hiPSC-derived cells to construct hair follicle organoids.

### Sweat gland organoid

Sweat glands originate from epidermal stem cells during embryonic development and consist of a duct segment and a secretory segment, surrounded by myoepithelial cells that aid in sweat secretion [61, 62]. Reprogramming epidermal cells into sweat gland-like cells has been demonstrated in mice [63]. Sun et al. [64] successfully converted human epidermal keratinocytes into sweat gland cells by overexpressing EDA and stimulating β2-adrenoceptors, leading to the establishment of human sweat gland organoids from the reprogrammed cells. These organoids were then transplanted onto mice, resulting in the generation of functional sweat glands in vivo. In another study, Yao et al. [65] utilized 3D bioprinting techniques to guide the differentiation of mesenchymal stem cells into mouse sweat glands and demonstrated the ability to repair damaged sweat glands in vivo after transplantation. Their research also identified the crucial roles of Hmox1 and CTHRC1 in sweat gland differentiation.

### Sebaceous gland organoid

Sebaceous glands are a vital component of the pilosebaceous unit and originate from a single progenitor cell cluster [66]. Mouse sebaceous gland organoids have been successfully generated using Blimp1^+^ cells isolated from adult mice [12] (Fig. 1d). In a complex skin organoid that mimics early organogenesis, sebaceous glands were observed after approximately 140 d of incubation, along with the presence of hair follicles [11]. However, the generation of human sebaceous gland organoids alone remains limited. One approach involves the use of immortalized cell lineages. Oulès and colleagues successfully generated sebaceous gland organoids using SebE6E7 sebocytes, and these organoids displayed a glandular portion coated by the ductal portion. They also investigated the role of GATA6 in the organoid model, concluding that GATA6 is crucial in sebaceous differentiation and proliferation by regulating the TGFβ signaling pathway [67]. While the cultivation of isolated sebaceous glands can contribute to the understanding of acne and other sebaceous gland-associated skin disorders [32], further research should aim to develop more complex structures that include other components of the pilosebaceous unit, such as hair follicles or even innervation [68].

### Skin tumor organoid

Skin cancer is a prevalent form of cancer worldwide, and gaining insight into its development is essential for the development of prevention and treatment strategies. The three major types of skin cancer are basal cell carcinoma, squamous cell carcinoma, and melanoma, while merkel cell carcinoma, sebaceous carcinoma, and melanoma are less common.

Cutaneous squamous cell carcinoma originates from the cells that make up the epidermis, making it feasible to replace normal keratinocytes with cancer cells when generating skin substitutes. Berning et al. [69] created a dermal equivalent based on fibroblast-derived matrix to support the growth of normal epidermis and seeded various types of cutaneous squamous cell carcinoma-derived cells onto it to establish a 3D tumor organoid. This model successfully replicated the invasive phenotype and matrix metalloproteinase secretion observed in tumor tissue in vivo. The tumor microenvironment plays a crucial role in tumorigenesis and tumor maintenance, necessitating an appropriate in vitro tumor model with complex tumor-matrix interactions. To preserve the heterogeneity of tumor cells and the microenvironment, Engelmann and co-workers placed fresh cutaneous squamous cell carcinoma tissue slices, approximately 3 mm thick, on top of the dermal equivalent and cultured them for up to 21 d [70]. As a result, this model maintained the heterogeneity of tumor cells and the microenvironment, including vital immune cells. This promising tool provides an opportunity to investigate the response of tumor tissue to treatments such as irradiation and targeted therapies. A 3D bioprinted model of cutaneous squamous cell carcinoma was fabricated using a skin substitute consisting of a 3D-printed fibroblast-embedded collagen-based dermis, a basement membrane layer, and an epidermal layer of normal keratinocytes [71, 72]. Although this model exhibited similar pathology, gene expression, and response to 5-fluorouracil treatment compared to tumor tissue in vivo, the manual pipetting of tumor cells onto the skin components impaired its consistency. To generate melanoma organoids, fresh tumor tissue obtained from patients was mechanically minced and enzymatically digested. The resulting spherical particles, ranging in size from 40 to 100 mm, were filtered and then resuspended in a solution of type I rat tail collagen [73]. These tumor organoids contained autologous lymphoid and myeloid cell populations and exhibited responsiveness to immune checkpoint blockade in short-term 3D microfluidic culture. Furthermore, immune-enhanced tumor organoids were generated by adding patient-derived lymph node cells [74]. To better preserve the histologic growth patterns and infiltrating immune cells in the organoids, a biopsy strategy based on fine-needle aspiration (FNA) was employed, which demonstrated superior results compared to traditional methods [75].

Forsythe et al. [76] successfully established patient-specific organoids for Merkel cell carcinoma by resuspending tissue specimens in a hydrogel and photocrosslinking them under ultraviolet light exposure. They also created immune-enhanced organoids by incorporating immunocompetent cells, including CD8^+^ cells, CD4^+^ cells, and antigen-presenting cells (APCs), obtained from matched patient whole blood or nodal lymph tissue. This 10-d culture strategy demonstrated both chemosensitivity and immunosensitivity, showcasing its potential to evaluate different treatment regimens and provide valuable insights for clinicians.

### Vascularized skin organoid

As organoids grow to a certain size, central necrosis becomes inevitable, particularly in parenchymal organ models like brain and liver organoids. This is due to the lack of an efficient vascular system for substance exchange. To replicate the in vivo structure of organs, it is crucial to recreate the interactions between the parenchyma and blood vessels. Therefore, vascularization is essential for long-term culture and the application of skin organoids in wound healing [76–78].

Vasculogenesis involves the self-assembly of vascular cells to form new blood vessels. Co-culturing with vascular cells has proven to be a reliable method for introducing blood vessels in various organoids, including liver organoids [79]. In Abaci’s study, an increase in hair follicle density resulted in significant necrosis and inhibited hair growth. To improve the dermis-like structure, human umbilical vein endothelial cells (HUVECs) were added to the type I collagen gel along with dermal fibroblasts at a ratio of 16:1. This resulted in the formation of capillary-like structures that became more organized and elongated after transplantation into mice [80]. Strunk et al. [81] generated spheroids consisting of endothelial colony-forming cells (ECFCs), fibroblasts, and keratinocytes derived from hiPSCs. Co-transplantation of endothelial progenitor cells accelerated vascularization and wound healing.

Growth factors such as FGF2 can facilitate angiogenesis. Xiong et al. [82] cultured 3D-printed skin organoids with scaffolds containing FGF2 and demonstrated that it promoted vascularization by recruiting endogenous cells after transplantation. VEGF, EGF, and PDGF are also incorporated into culture strategies due to their crucial effects on the natural wound healing process, although it remains to be seen whether they contribute to skin organoid angiogenesis [83]. In a recent study, attempts were made to introduce blood vessels into hiPSC-derived skin organoids using a self-assembly method [81]. The researchers transfected stabilized KGF mRNA and FGF-7 mRNA into hiPSCs to enhance keratinocyte differentiation and fitness, and used PDGFs in human platelet lysate to improve organoid proliferation and graft angiogenesis.

**Fig. 2:**
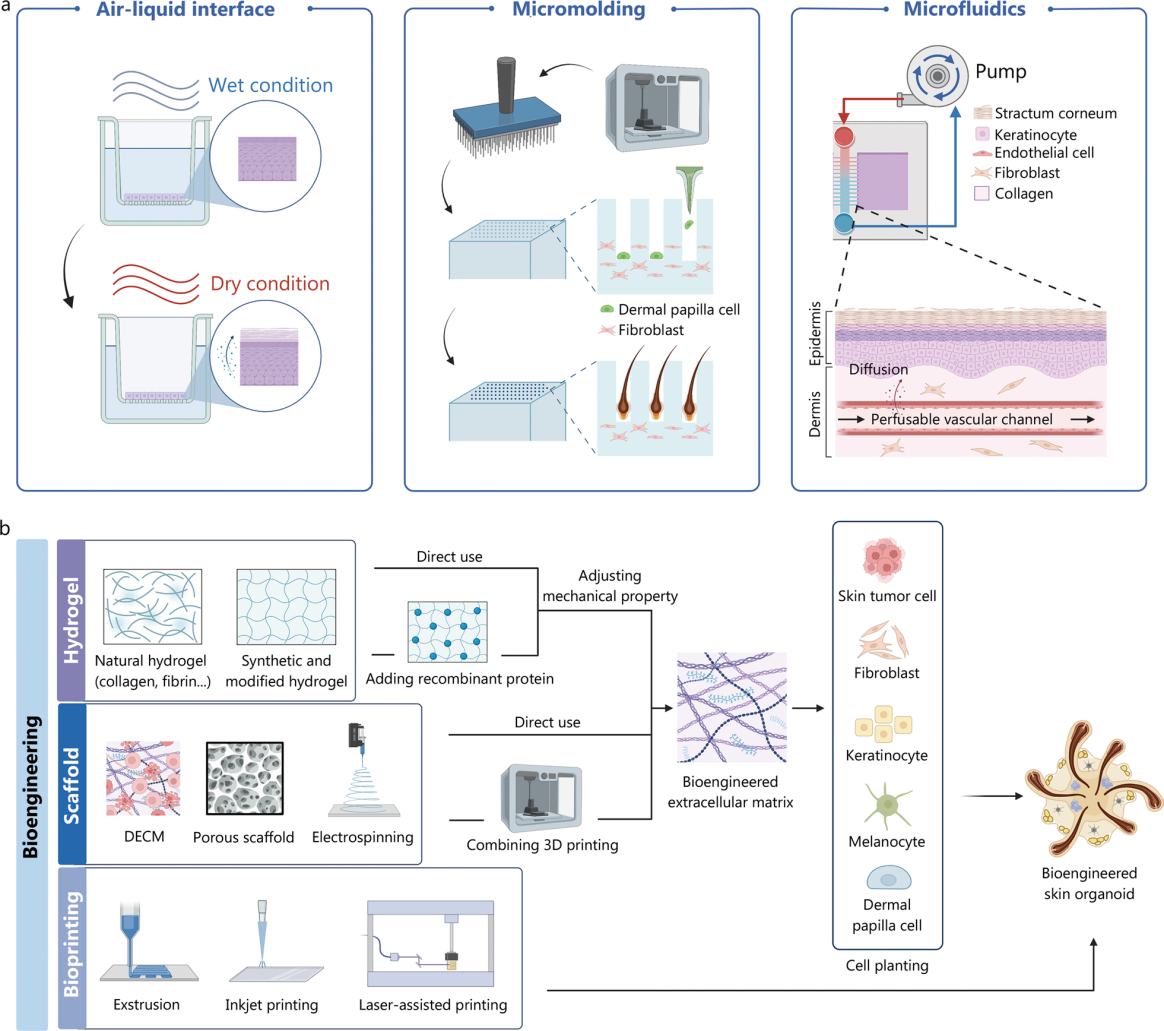
Widely applied biomaterials and bioengineering strategies. **a** The construction of an appropriate extracellular matrix (ECM) is essential for the maturation of skin organoids, and this can be achieved using natural or artificial hydrogels and various scaffolds. Additionally, 3D printing technology can be employed to recreate the solid structures of the stratum corneum or basement membrane, allowing for precise spatial arrangement of cells. **b** Three bioengineering strategies have been developed to regulate the microenvironment and microstructure of skin organoids with precision. The air–liquid interface approach intervenes in the gas-phase environment and promotes keratinocyte differentiation. Microplasticity enables the correct and orderly arrangement of different intercellular compartments. Microfluidics provides artificial conduits for endothelial growth and allows for precise control over the timing and quantification of material input to the system. DECM decellularized extracellular matrix

Microfluidic systems have been employed to simulate blood vessels and enhance vessel formation and perfusion in various types of organoids, including kidney and skin [84–86] (Fig. 2a). Mori et al. [87] fabricated perfusable vascular channels coated with endothelial cells within a cultured skin-equivalent. These artificial vessels can serve as pathways for delivering nutrients or as models for fabricated vascular networks. Skin organoids on a chip have also contributed to enhanced barrier properties and improved phenotypic differentiation. However, microfluidic devices have not yet been able to fully replicate capillaries in organoids [88].

## Bioengineering strategies

The limited reproducibility of the organoid model is a challenging aspect that can be addressed by implementing a controlled environment. In order to gain better control over the organization and functionality of stem cell-derived organoids, researchers have turned to bioengineering solutions. These solutions show great potential in enhancing maturation and reducing the need for xenogeneic materials [89]. Among these approaches, biomaterials-enhanced 3D bioprinting methods offer the ability to achieve more precise architectures and improve cellular placement compared to traditional models. Moreover, they provide a high-throughput and reproducible platform for drug screening and toxicity testing (Fig. 2b).

### Hydrogel

Matrigel, derived from the secretions of Engelbreth-Holm-Swarm mouse sarcoma cells, is commonly used as a culture environment for organoids and has been shown to promote the maturation of skin appendages, including hair follicles and sweat glands [90, 91]. However, the biochemical properties of different batches of Matrigel can vary, leading to poor reproducibility. To overcome this challenge, researchers have been exploring well-defined alternatives to this complex material, such as natural or synthetic hydrogels [92, 93]. Collagen gels, which mimic the natural environment for fibroblasts, have been found to induce differentiation in various types of organoids and may be suitable for dermis culture [94]. However, processed collagen has weak mechanical properties, making it inherently unstable [95]. Modified gelatin derivatives, such as gelatin methacryloyl (GelMA), have shown improved degradability and stability. Barros et al. [96] developed a 3D skin model using GelMA, incorporating multilayered keratinocytes, dermal fibroblasts, and endothelial cells. Tan et al. [97] discovered that GelMA reduced the exfoliative behavior of keratinocytes due to its high attachment properties. Moreover, glycosaminoglycans (GAGs), fibrin, and other hydrogels have been integrated into skin equivalents due to their beneficial biological properties.

### Scaffolds

Scaffold design is based on a comprehensive understanding of the extracellular matrix (ECM), which plays a pivotal role in biological adhesion, receptor signaling, cell survival, and morphogenesis (Fig. 2b). Scaffolds hold immense potential in skin tissue engineering and the culture of skin organoids. The decellularization of whole organs has been successfully applied in the heart, liver, kidney, and lung. Decellularized ECM (dECM) provides an optimal non-immunogenic microenvironment with natural 3D structures and various adhesion components [98]. DECM scaffolds can repair and regenerate skin tissues by preserving physical signals that promote keratinocyte adhesion and the growth of angiogenic cells [99, 100]. Hansmann et al. [101] successfully generated a vascularized skin equivalent by seeding cells on a decellularized segment of porcine jejunum. Numerous studies have demonstrated the remarkable potential of dECM in rapidly recapitulating organ function.

Inert scaffolds offer unique advantages. Porous scaffolds can take various forms, including sponges, foams, meshes, and biodegradable fibers. The ideal porous scaffold possesses a specific pore size, high porosity, and an appropriate surface-to-volume ratio, enabling the diffusion of nutrients and drugs. Furthermore, it must be biocompatible, biodegradable, and non-toxic to cells and the body [102–104]. Roger et al. [105] created a dermal construct with human fibroblasts that secrete ECM proteins using an inert porous scaffold to avoid the use of animal-derived materials. Electrospinning is utilized to produce fibrous scaffolds, often coated with adhesive proteins. Electrospun poly l-lactic acid (PLLA) fiber is a commonly used scaffold in tissue engineering and the generation of skin organoids. Girija et al. [106] biofunctionalized the PLLA scaffold with collagen to enhance cell interactions. Adhesion and migration of seeded keratinocytes and fibroblasts were observed after 10 d.

### Bioprinting

3D printing technology enables the creation of solid architectures to precisely regulate the spatial arrangement of cells [107]. Abaci et al. [80] utilized 3D printing molds to cast type I collagen gel with dermal fibroblasts, mimicking the dermis with follicles. Skin organoids were generated by seeding dermal papilla cells (DPCs) in microwell gels of varying densities. Keratinocytes were then added to fill the microwells and serve as the epidermal component. After three weeks of generation, they observed keratinocyte differentiation and the expression of hair lineage markers. In some constructs, hair fibers elongated and spontaneously rearranged their positions from a right angle to an obtuse angle.

There are three primary strategies for bioprinting: extrusion, inkjet, and laser-assisted printing [107]. Cubo et al. [108] utilized extrusion printing modules consisting of four tunnels to combine human plasma, human fibroblasts, calcium chloride, and human keratinocytes. The resulting equivalents were allowed to differentiate for 17 d both in vitro and in vivo, exhibiting a stratum corneum and basement membrane structure. This technique enables printing a high density of cells but is limited by shear stress. Laser-assisted bioprinting was also employed to create cellularized skin substitutes capable of recapitulating rete ridge-like structures, resulting in a significant improvement in basement membrane formation and promoting epidermal proliferation and differentiation [48, 109].

## Applications and prospects

Organoids offer a robust platform that allows researchers to manipulate cell populations and cellular environments artificially, catering to the study of a diverse range of physiological and pathological contexts. This holds great potential for investigating skin developmental biology, pathology, and clinical applications [110, 111].

These application areas highlight the versatility and potential of skin organoids in advancing research and clinical applications in the field of skin biology.

### Developmental biology

**Fig. 3:**
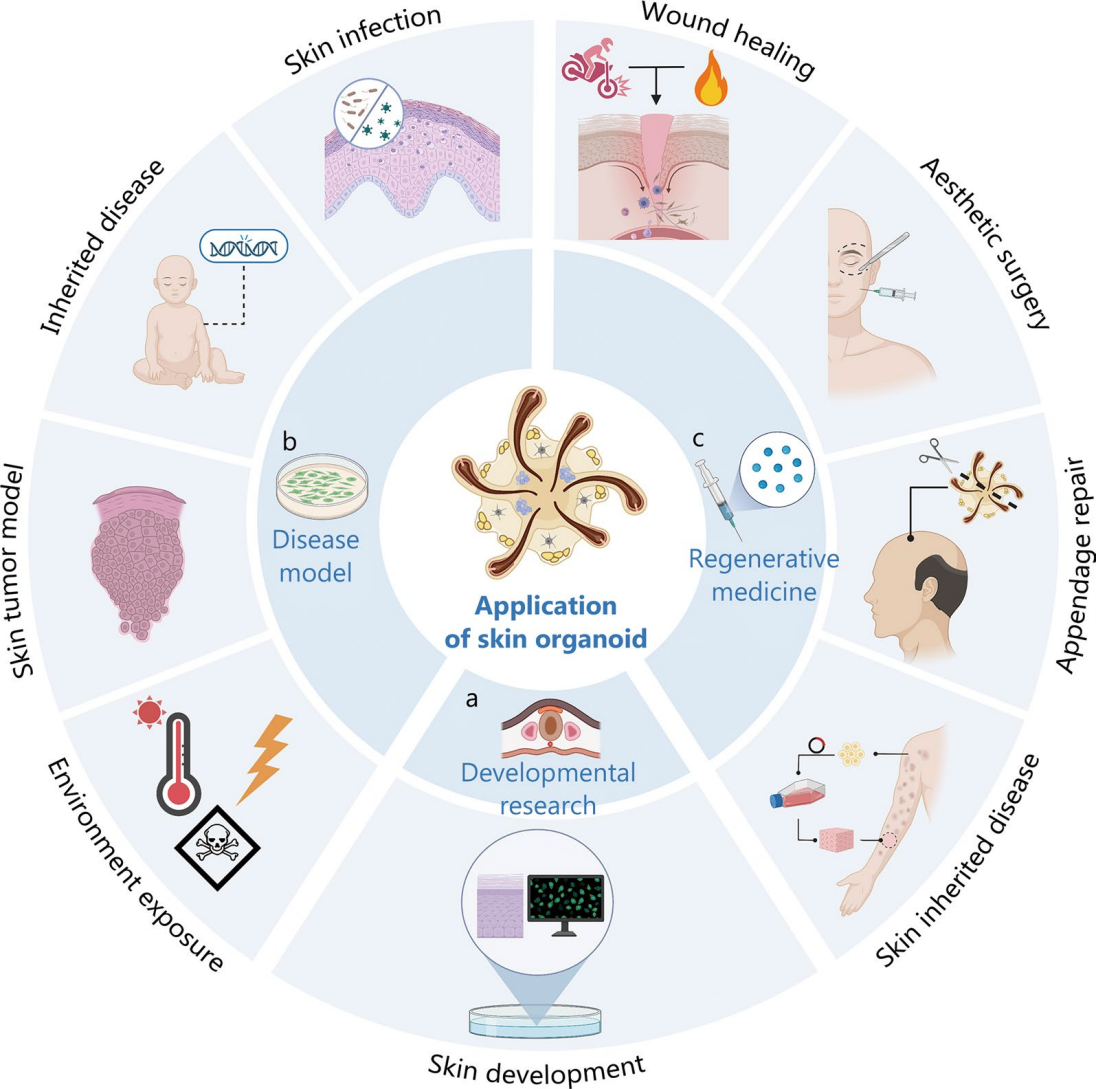
The application of skin organoid. There are three primary application areas of skin organoids, each with representative works. **a** Developmental research: skin organoids provide an opportunity to investigate the impact of chemical signaling on skin maturation. This field enables researchers to delve into the mechanisms that govern the development of the skin. **b** Disease modeling: skin organoids serve as valuable model systems for studying various skin infections such as atopic dermatitis, inherited skin diseases, skin cancers, and environmental exposures including ionizing radiation and chemicals. They offer a platform for understanding disease mechanisms, developing treatments, and conducting drug screening. **c** Regenerative medicine: skin organoids offer insights into the pathophysiology of wounds resulting from surgery, trauma, or burns. They also hold promise for applications in aesthetic surgery for facial repair and for treating conditions like alopecia that involve the loss of skin appendages. The use of patient-derived skin organoids in a 3D culture system has been explored for the therapy of inherited diseases

Access to human fetal tissue for laboratory investigation is limited due to ethical and regulatory challenges, which has hindered our advanced understanding of human skin development. Currently, research heavily relies on rodent models that do not fully replicate the features of human embryogenesis, resulting in significant gaps in our knowledge about human skin development. However, skin organoids offer a valuable resource for studying skin organogenesis, providing ample materials to investigate early human skin development and overcome the limitations of current research methods (Fig. 3a).

A promising avenue for future research is to explore the influence of the microenvironment and cell-to-cell interactions on stem or progenitor cells during skin development, including the intricate process of skin appendage induction, such as hair follicle formation. This process involves various signaling pathways, including Wnt, FGF, and BMP, which govern the early differentiation of ectodermal and mesenchymal cell lineages [18]. While the role of developmental signaling cues has been extensively studied in mice, there is a paucity of research directly examining these cues in human skin development [18, 40, 112, 113]. Additionally, investigating the mechanical cues and their interplay with chemical signaling in skin maturation could be pursued by incorporating muscle into the system [114]. Cell migration is also a critical aspect of skin development, and understanding the fate of different lineages, such as melanocytes derived from neural crest cells, and their interaction with the environment during embryonic migration is crucial [22]. Pigmented hair organoids containing melanocytes provide an excellent model for studying the migration and maturation of this lineage [11].

Replicating the specific characteristics of different regions of human skin in skin organoid cultures represents an exciting avenue for embryonic development research. The human body exhibits diverse skin types that are specialized to specific anatomical locations. For instance, the skin on the soles of the feet is thicker and contains numerous sweat glands, while the skin on the eyelids and lips is thinner and lacks skin appendages. This regional heterogeneity is closely tied to dermis and substratum development, and further investigation using organoid models is necessary to enhance our understanding of these processes [113].

Furthermore, conducting comparative studies between human organoids and animal models, as well as organoids derived from animal models, can offer valuable insights into the developmental similarities or differences between humans and other species. For instance, hair follicle organoids have been successfully created using both mouse and human-induced pluripotent stem cells [11, 115]. Extensive research can be conducted to explore the developmental and pathological features of these two types of organoids, providing additional evidence to either support or challenge the conclusions drawn from studies utilizing rodent models.

### Disease modeling

Organoids offer a versatile tool for investigating the structural and cellular changes in human skin under various conditions, such as exposure to genotoxic substances, invasion by pathogenic microorganisms, or rare gene mutations. This enables detailed studies on the effects of different interventions at a cellular level. Moreover, the use of patient-derived cells in skin organoids allows for personalized therapies and drug screening, making them highly valuable in clinical applications [116]. Looking ahead, the development of complex 3D constructs and personalized scaffolds tailored to individual patient wounds holds great potential for customized treatment regimens [117] (Fig. 3b).

#### Infectious diseases

The skin is susceptible to microbial infections as it acts as the primary barrier of the human immune system. hiPSC-derived organoids cultivated using the air–liquid interface (ALI) method serve as an effective model to mimic atopic dermatitis. By activating Wnt signaling, this culture method forms a stratified squamous epithelial structure similar to human skin and allows for the colonization and infection of *S. aureus*, mimicking the conditions of atopic dermatitis. This model provides a direct link between atopic dermatitis and *S. aureus* colonization and infection, making it valuable for evaluating the efficacy of novel therapies [118]. Additionally, hair-bearing skin organoids have been employed to study hair loss in COVID-19 patients. Research has shown that SARS-CoV-2 can infect KRT17^+^ hair follicles and directly affect both hair follicle and neuronal development in the skin, leading to impaired hair follicle and epidermal growth [58].

#### Inherited diseases

Although inherited skin diseases may be rare, their severity necessitates a comprehensive understanding of their underlying mechanisms and the development of effective treatments. In the case of psoriasis, research has validated the therapeutic effect of a GLUT inhibitor on human skin organoids, offering a potential new strategy for managing this condition [119]. A 3D culture system utilizing hiPSC-derived epithelial and mesenchymal (EM) organoids has been introduced for localized scleroderma (LoS) therapy. This approach has shown promising results in reducing skin fibrosis in scleroderma-affected skin [120]. For junctional epidermolysis bullosa (JEB), combined ex vivo cell and gene therapies have successfully regenerated autologous transgenic keratinocytes, resulting in a fully functional epidermis and providing a novel therapeutic avenue for this disease [121]. Patient-specific iPSC-derived skin organoids have proven to be an effective platform for high-throughput drug screening. In a study related to systemic sclerosis (SSc), screening with patient-derived organoids identified selective estrogen receptor modulator (SERM)-class drugs as potential candidates for treating SSc fibrosis [46].

#### Skin tumor

Organoids have been used to recapitulate several types of skin cancers, including squamous cell carcinoma, melanoma, and Merkel cell carcinoma, as described above, providing valuable insight for basic research and clinical care. These tumor models have been able to replicate invasion phenotypes and model the tumor microenvironment, advancing research into tumor induction and development [69, 122]. As individual differences and tumor heterogeneity can impact patient-specific treatment responses, patient-derived organoids have potential for personalized therapy. They can be used to monitor patient immune responses, tumor viability, and drug sensitivity, helping physicians determine the best quality regimen, particularly for refractory tumors.

#### Environmental exposure

Skin organoids offer a valuable platform for investigating the previously unknown effects of environmental factors on the skin. Researchers have utilized 3D skin organoids derived from hiPSC-derived keratinocytes to study the impact of ionizing radiation (IR) exposure and DNA damage response [44]. The findings revealed a decrease in DNA damage response, including DNA repair activity, during differentiation. In another study, low-dose γ-irradiation was applied to skin substitutes prior to transplantation onto nude mice, resulting in focal dysplasia in xenografted epidermises and exhibiting characteristics of epithelial-to-mesenchymal transition (EMT). This study highlighted that even minimal radiation stress during the regeneration of keratinocyte stem and precursor cells can create a microenvironment that may promote long-term carcinogenesis [42]. Additionally, investigating the effects of environmental factors, such as air pollution and cigarette smoking, on skin aging and related diseases is both feasible and necessary using in vitro treated skin organoids. The use of organoids provides a standardized tool for identifying related signaling pathways and gaining insights into the underlying mechanisms involved [123].

### Regenerative medicine

Skin injuries resulting from surgery, trauma, or burns can have significant physiological and psychological impacts. Skin organoids offer a promising avenue for gaining deeper insights into the pathophysiology of challenging-to-heal wounds and the permanent loss of skin appendages. Moreover, they hold potential as a cell source for cell therapies and skin transplantation, making them viable candidates for autografting procedures and epithelial reconstitution surgeries [124] (Fig. 3c).

Conventional facial skin transplantation often involves harvesting tissue from other body parts, which can lead to scarring and immune rejection due to differences in function and composition compared to facial tissue [125]. In recent years, the use of skin organoids derived from hiPSCs for facial repair and skin reconstruction has been proposed, and significant progress has been made in producing skin organoids that can seamlessly integrate with mouse skin [81]. This approach effectively reduces scar formation and mitigates the issue of immune rejection, providing a valuable tool in the field of regenerative medicine [81, 126]. In 2023, Pappalardo et al. [127] reported a groundbreaking study on the creation of wearable edgeless skin constructs using 3D printing, which minimizes the need for suturing and improves the effective coverage of wounds.

Skin wounds are highly sensitive to even minor temporal changes in various cytokines and non-coding RNAs, which can have a significant impact on wound healing [41, 117]. Skin organoids have provided valuable in vitro models for studying wound healing, allowing researchers to investigate biophysical and biochemical cues without the need for costly and time-consuming animal studies. These mechanistic studies offer advantages that will greatly contribute to the clinical translation of organoids [128]. Importantly, these skin organoids are capable of releasing bioactive substances related to wound healing in a controlled manner and can recreate the intricate cell–cell and cell-extracellular matrix interactions of stem/progenitor cells. This capacity allows these 3D constructs to meet the necessary criteria for clinical applications [110].

Alopecia is a widespread condition that affects both males and females worldwide, significantly impacting their physical appearance. Fortunately, skin organoids present a promising solution for treating alopecia and restoring natural hair growth. The latest generation of hiPSC-derived organoids, which include hair follicles, can be developed for use in follicular unit transplantation [11]. This technique enables the production of new hair shafts that match the original donor site, avoiding potential harm associated with conventional hair transplantation in other areas of the scalp. Additionally, skin organoids can be genetically modified to enhance their survival and functionality in the recipient site, such as by reducing androgen receptors.

## Limitations

It is important to acknowledge the limitations of organoids, as they have hindered the development of their applications in disease modeling and clinical medicine. However, these challenges also provide valuable directions for further research.

While 3D organoids serve as useful in vitro skin models, they have certain limitations that restrict their utility in pathological studies of multiple organ systems. These limitations include a lack of normal inter-tissue communication, incomplete development of complex vasculature, and difficulties in establishing complex neural networks and immune cell clusters [20, 129]. Moreover, skin organoids derived from hiPSCs only mimic early skin structures in the fetus and more mature structures after prolonged culture. They are not effective at modeling the intricate and dynamic changes that occur during aging and in vitro rejuvenation of senescent skin. These limitations highlight the heavy reliance of current skin organoid cultures on artificial nutritional and signaling support, underscoring their incomplete maturation [14].

Future studies can address these limitations by integrating microfluidic devices to mimic in vivo signaling centers and concentration gradients. This approach would enable better in vitro guidance and spatiotemporal control of skin organoid growth and self-organization, ultimately advancing organotypic cultures to the level of in vitro organ-on-a-chip systems. Additionally, in vivo transplantation of skin organoids into murine hosts can further promote their maturation [14, 41, 110, 117, 129, 130]. Integration with multi-organ chips can also be explored to establish connections and communication between skin organoids and other preformed organoids [14, 84, 86]. Another approach to enhance the size and complexity of skin organoids is to incorporate angiogenesis-associated endothelial cells, peripheral nervous tissue stem cells, hematopoietic stem cells, and their corresponding microenvironments in co-culture systems. This would further establish the complex vasculature and neural networks within the organoids [41, 110, 129].

It is worth noting that there is currently no standardized protocol for constructing skin organoids worldwide [111]. Organoids relying on self-organizing principles often exhibit high heterogeneity, which presents a challenge in establishing standardized building materials and ensuring precise quality control and co-culture of cells. To address these challenges, the research strategy described above offers a potential solution by exploring stepwise procedures for skin organoid establishment and creating organoid biobanks for different pathologies [14].

A significant focus of current studies is to address the issue of immune rejection associated with organ transplantation, particularly in the context of hiPSC-derived organoids [81]. Immune rejection has limited the clinical application of skin transplantation [131]. Conventional strategies for large organ transplantation involve lifelong immunosuppressant treatment, which compromises the immune system and increases the risk of microbial infection and tumorigenesis. As a result, progress in this area has been slow and cautious [132]. However, hiPSC-derived skin organoids have the potential to overcome the problem of immune rejection, as they are theoretically non-immunogenic and suitable for large-scale autografts [129]. Future studies must carefully evaluate the safety and efficacy of autologous stem cell transplantation of skin organoids to address the immune rejection issue [81]. This will ensure that the advantages of hiPSC-derived organoids are fully utilized while effectively addressing immune rejection associated with organ transplantation.

The skin contains various immune cells, including Langerhans cells, dermal dendritic cells, and macrophages, which not only play a crucial role in combating infectious diseases but also contribute to the normal homeostasis of the skin. Therefore, incorporating immune components into skin organoid models significantly enhances their reliability and credibility. Previous studies have utilized hiPSC-derived skin organoids to model viral and bacterial infections [58, 118]. While these investigations successfully replicate the physical barrier function of the skin, the absence of essential immune components may compromise the relevance of these models. It is therefore crucial to include immune components in skin organoid model systems. For example, autoimmune skin disorders such as psoriasis involve intricate interactions between immune cells and non-immune cells, with unclear underlying mechanisms [133]. Consequently, an ideal organoid platform that includes immune components has the potential to accelerate advancements in uncovering the underlying mechanisms and developing novel therapeutics. In the context of skin tumor organoids, co-culturing patient-derived immune cells with the tumor organoid has been employed [74, 76]. This approach creates a more authentic model that faithfully mimics the interaction between tumor cells and the immune system. Overall, these co-culture strategies provide reliable protocols for the long-term maintenance of immune cells within skin organoid systems, facilitating groundbreaking studies of skin diseases [134].

The realization of personalized therapy necessitates the establishment of a population-based large-scale stem cell organoid bank for emergency management of severe burns. However, the personalized construction of organoids is currently limited by the high manufacturing costs, which hinders their applicability in precision therapy and makes it challenging to implement them in less-developed regions [135]. These challenges stem from inadequate expansion rates of stem cell populations, complex processes of directed differentiation into skin tissue, and substantial artificial intervention in terms of trophic and signaling support to the system [136]. To overcome these challenges, future strategies could involve the development of a new generation of standardized high-throughput construction techniques that reduce the costs of large-scale production, thereby making skin organoids more accessible for research and clinical applications [14, 41].

## Conclusions

Skin organoids are emerging as a promising modelling strategy that drives advancements in healthcare, particularly in the fields of disease modelling and regenerative medicine. With continuous technical improvements, the culture system of skin organoids has matured, allowing them to progress from simple in vitro cultures to complex systems that encompass the epidermis, dermis, and appendages. The development of numerous skin organoids with diverse appendages and distinct phenotypes has provided a convenient and high-quality platform for studying skin development, microbial infections, inherited skin diseases, and neoplasms. These advantages not only lay a solid foundation for the clinical application of skin organoids in regenerative medicine and drug screening but also create opportunities for precision medicine and personalized treatment strategies. Although challenges persist, given the rapid technological advancements in the field, we are confident that skin organoid systems will continue to overcome their limitations and offer unprecedented opportunities to enhance human skin health.

## Data Availability

Not applicable.

## Abbreviations

2D/3D: Two dimensional/three dimensional
AD: Human atopic dermatitis
ALI: Air liquid interface
APC: Antigen presenting cell
Blimp1: B lymphocyte induced maturation protein 1
BMPs: Bone morphogenetic proteins
CEA: Carcinoembryonic antigen
CNCC: Cranial neural crest cell
CTHRC1: Collagen triple helix repeat containing 1
DECM: Decellularized extracellular matrix
DPCs: Dermal papilla cells
DPP4/CD26: Dipeptidyl peptidase-4
ECFCs: Endothelial colony-forming cells
ECM: Extracellular matrix
EDA: Ectodysplasin A
EGF: Epidermal growth factor
EM: Epithelial and mesenchymal
EMT: Epithelial-to-mesenchymal transition
ESCs: Embryonic stem cells
FGFs: Fibroblast growth factors
FLG: Filaggrin
FMO1: Flavin-containing monooxygenase 1
FNA: Fine-needle aspiration
GAGs: Glycosaminoglycans
GelMA: Gelatin methacryloyl
GLUT: Glucose transporter
HA: Hyaluronic acid
HF: Hair follicle
HFSCs: Hair follicle stem cells
HLA: Human leukocyte antigen
Hmox1: Heme oxygenase 1
hTERT: Human telomerase reverse transcriptase
HUVECs: Human umbilical vein endothelial cells
iPSCs: Induced pluripotent stem cells
JEB: Junctional epidermolysis bullosa
KRT: Keratin
KGF: Keratinocyte growth factor
LEF1: Lymphoid enhancer-binding factor 1
LSP1: Lymphocyte specific protein 1
OMM: Organoid mature medium: Dmem + neurobasal + n2 + (b27-vitamina) + mercaptoethanol + normocin
PDGF: Platelet derived growth factor
PDGFR: Platelet derived growth factor receptor
PLLA: Poly l-lactic acid
RA: Retinoic acid
SebE6E7: Cell line name
SERM: Selective estrogen receptor modulator
SFRP2: Secreted frizzled-related protein 2
SHH: Sonic hedgehog
SMAD: Drosophila mothers against decapentaplegic protein
SSc: Systemic sclerosis
SV40Tt: Simian virus 40 large t and small t antigen
TGF-β: Transforming growth factor β
TP63: Tumor protein p63
